# Supplementary material for: Integrative Taxonomy of Armeria arenaria (Plumbaginaceae), with a Special Focus on the Putative Subspecies Endemic to the Apennines
Source: Biology (Basel). 2022 Jul 14;11(7):1060. doi: 10.3390/biology11071060 (PMC9312046; doi:10.3390/biology11071060)
Supplement: Supplementary file 1 [file biology-11-01060-s001.zip › biology-1779446-supplementary.pdf]

Supplementary Materials

Table S1. Shape of the leaf apex according to the second grouping hypothesis. A figure on the right is provided for comparison. Significance according to Fisher's exact test.

SUM_LEAF_APEX	FB	marginatoid	arenarioid (FB excluded)
acute (A)	0	60	9
cucullate (B)	23	10	123
significance	a	b	a

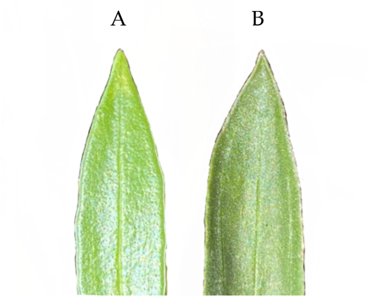

Table S2. Presence of teeth on summer leaf margins according to the second grouping hypothesis. Leaf margins are always hyaline, but in some cases uni- or pluri-cellular teeth emerge from the margin. Significance levels according to Fisher exact test.

MAR_SUM_LEAF	FB	marginatoid	arenarioid (FB excluded)
no teeth	1	71	105
teeth	22	3	27
significance	a	b	c

Table S3. Number of veins on summer leaves based in cross section, according to the second grouping hypothesis. Significance levels according to Fisher exact test.

N_ SUM_VEINS	FB	marginatoid	arenarioid (FB excluded)
3	4%	88%	5%
5	48%	12%	29%
7	48%	0%	60%
9	0%	0%	4%
10	0%	0%	2%
individuals (n)	23	74	129
Significance	a	b	a

Table S4. Mean and standard deviation values (mm) of quantitative characters for the twelve populations studied. Characters as in Table 2 and populations as in Table 1. * = type locality.

Character	AA	BO	BR	FB*	GA	LA*	LL*	MB*	MC	MP	PS	TV
ANG_SUM_TIP	11.79 ± 3.95	13.71 ± 5.62	20.55 ± 7.03	16.96 ± 6.16	17.33 ± 7.41	10.8 ± 4.51	15.9 ± 6.51	15.55 ± 4.54	13.3 ± 4.0	21 ± 7.49	9.7 ± 2.98	17.6 ± 6.4
ANG_WIN_TIP	18.15 ± 3.13	34.38 ± 7.8	33.8 ± 8.4	21.85 ± 6.76	31.67 ± 11.08	19.85 ± 4.9	26.35 ± 9.9	26.68 ± 6.55	18.5 ± 4.27	31.13 ± 15.07	N/A	32.27 ± 11.18
AWN_LENG	0.76 ± 0.11	1.02 ± 0.17	0.95 ± 0.16	1.04 ± 0.19	1.08 ± 0.2	0.82 ± 0.13	0.87 ± 0.18	0.76 ± 0.2	0.91 ± 0.18	0.93 ± 0.21	1.01 ± 0.35	0.95 ± 0.21
DIAM_CAP	17.95 ± 2.04	16.46 ± 1.63	17.29 ± 1.58	13.85 ± 1.5	15.53 ± 0.96	19.18 ± 1.27	15.59 ± 1.19	19.08 ± 1.41	18.7 ± 1.43	15.23 ± 1.89	18.48 ± 2.25	17.98 ± 1.4
HEIGTH	327.5 ± 65.01	430.71 ± 80.76	430.95 ± 74.03	446.57 ± 109.38	452.28 ± 76.51	300.8 ± 45.82	498.8 ± 74.42	318.15 ± 61.72	252.65 ± 52.32	340.75 ± 62.72	429.3 ± 59.19	446.2 ± 55.01
LENG_CAL_PED	1.99 ± 0.64	1.3 ± 0.43	1.83 ± 0.58	1.41 ± 0.6	1.24 ± 0.3	2.18 ± 0.48	1.76 ± 0.48	2.19 ± 0.71	1.86 ± 0.69	1.31 ± 0.31	2.26 ± 0.56	1.46 ± 0.5
LENG_CAL_TUBE	3.45 ± 0.24	3.37 ± 0.28	3.1 ± 0.36	2.84 ± 0.22	3.3 ± 0.28	3.36 ± 0.23	3.14 ± 0.19	3.27 ± 0.37	3.16 ± 0.35	3.02 ± 0.29	3.42 ± 0.31	3.36 ± 0.27
LENG_INNER_INV_BRACT	7.73 ± 0.83	7.09 ± 0.77	6.95 ± 0.69	5.29 ± 0.91	6.37 ± 0.76	7.9 ± 1.19	6.87 ± 0.77	9.29 ± 0.9	7.95 ± 1.06	6.39 ± 0.96	7.62 ± 0.8	6.64 ± 0.89
LENG_INNER_SPI_BRACLE	6.42 ± 1.16	4.71 ± 0.67	4.8 ± 0.52	4.01 ± 0.52	3.68 ± 0.61	5.93 ± 1.02	4.55 ± 0.62	5.79 ± 1.0	5.72 ± 1.28	4.15 ± 0.52	5.37 ± 0.75	4.65 ± 0.87
LENG_INNER_SPI_BRACT	8.76 ± 0.92	7.09 ± 0.61	7.41 ± 0.67	6.0 ± 0.61	6.36 ± 0.49	8.7 ± 0.84	6.9 ± 0.53	8.81 ± 0.81	8.67 ± 1.22	6.46 ± 0.86	7.65 ± 0.84	7.44 ± 0.76
LENG_INTER_INV_BRACT	7.31 ± 0.99	6.63 ± 0.98	5.97 ± 0.68	3.96 ± 0.81	5.73 ± 0.47	7.53 ± 1.27	5.75 ± 0.76	8.77 ± 1.29	7.28 ± 1.43	5.83 ± 1.03	6.79 ± 0.77	5.68 ± 0.94
LENG_OUT_INV_BRACT	7.46 ± 1.58	10.92 ± 4.32	8.56 ± 1.75	10.4 ± 6.78	7.83 ± 1.72	6.8 ± 1.55	8.77 ± 1.85	7.06 ± 1.87	6.33 ± 1.33	9.22 ± 3.51	7.35 ± 1.62	6.88 ± 1.41
LENG_OUTER_SPI_BRACLE	6.17 ± 0.97	4.6 ± 0.49	4.62 ± 0.54	4.03 ± 0.39	3.54 ± 0.57	5.56 ± 0.95	4.68 ± 0.41	5.64 ± 0.93	5.76 ± 1.12	4.04 ± 0.61	5.25 ± 0.76	4.7 ± 0.76
LENG_OUTER_SPI_BRACT	8.38 ± 0.79	6.8 ± 0.54	7.04 ± 0.58	5.4 ± 0.52	6.06 ± 0.57	8.21 ± 0.63	6.48 ± 0.48	8.4 ± 0.69	8.12 ± 0.91	5.93 ± 0.83	7.17 ± 0.95	7.18 ± 0.73
LENG_SUM_LEAF	71.56 ± 32.74	111.16 ± 27.2	95.52 ± 23.61	84.23 ± 28.95	102.28 ± 24.39	70.85 ± 21.05	102.28 ± 24.82	82.17 ± 39.33	61.71 ± 25.92	79.68 ± 27.34	112.45 ± 38.1	98.21 ± 25.52
LENG_WIN_LEAF	39.95 ± 13.04	46.6 ± 13.57	60.71 ± 27.76	50.63 ± 31.29	61.58 ± 11.35	43.76 ± 17.55	52.37 ± 24.44	35.31 ± 11.36	30.36 ± 9.58	36.23 ± 10.95	N/A	46.65 ± 19.82

Character	AA	BO	BR	FB*	GA	LA*	LL*	MB*	MC	MP	PS	TV
LIMB_LENG	2.65 ± 0.21	2.14 ± 0.21	2.27 ± 0.24	1.81 ± 0.26	1.92 ± 0.21	2.44 ± 0.18	2.07 ± 0.17	2.79 ± 0.26	2.73 ± 0.31	1.75 ± 0.28	2.17 ± 0.27	2 ± 0.32
SCA_DIAM	1.08 ± 0.14	1.5 ± 0.25	1.27 ± 0.18	1.4 ± 0.33	1.21 ± 0.18	1.57 ± 0.35	1.66 ± 0.21	1.24 ± 0.23	1.35 ± 0.26	1.28 ± 0.15	1.44 ± 0.23	1.42 ± 0.23
SCA_LENG	305.29 ± 71.96	399.14 ± 70.96	404.75 ± 78.11	406.13 ± 113.29	430.61 ± 75.23	266.63 ± 39.09	485.15 ± 66.09	292.8 ± 73.63	213.75 ± 47.96	281.35 ± 80.97	402.75 ± 73.02	427.5 ± 60.86
SHEATH_LENG	17 ± 2.5	34.34 ± 6.01	28.92 ± 4.69	21.24 ± 10.47	24.93 ± 7.5	19.32 ± 4.06	33.68 ± 7.24	16.49 ± 4.19	14.84 ± 3.38	23.48 ± 6.85	24.93 ± 3.9	28.22 ± 5.24
WIDTH_CAL_TUBE	1.07 ± 0.15	1.08 ± 0.14	0.98 ± 0.14	0.72 ± 0.15	1.01 ± 0.15	1.14 ± 0.15	0.88 ± 0.11	0.99 ± 0.16	1.11 ± 0.17	0.97 ± 0.12	1.02 ± 0.18	1.11 ± 0.15
WIDTH_IAL_SUM	0.08 ± 0.02	0.09 ± 0.02	0.07 ± 0.02	0.07 ± 0.02	0.08 ± 0.02	0.12 ± 0.02	0.06 ± 0.02	0.1 ± 0.02	0.11 ± 0.02	0.09 ± 0.02	0.07 ± 0.02	0.06 ± 0.01
WIDTH_IAL_WIN	0.12 ± 0.07	0.11 ± 0.02	0.1 ± 0.03	0.09 ± 0.02	0.09 ± 0.02	0.13 ± 0.03	0.08 ± 0.02	0.12 ± 0.02	0.12 ± 0.02	0.09 ± 0.02	NA	0.08 ± 0.02
WIDTH_INNER_INV_BRACT	4.19 ± 0.78	4.78 ± 0.88	4.17 ± 0.75	3.7 ± 0.62	3.67 ± 0.88	5.04 ± 0.91	4.08 ± 0.62	5.02 ± 0.72	4.15 ± 1.11	4.61 ± 0.98	3.69 ± 0.57	4.65 ± 0.62
WIDTH_INNER_SPI_BRACLE	2.51 ± 0.55	3.01 ± 0.44	2.32 ± 0.3	2.35 ± 0.3	2.38 ± 0.51	2.53 ± 0.48	2.78 ± 0.42	2.68 ± 0.76	2.16 ± 0.8	2.59 ± 0.43	2.74 ± 0.52	2.86 ± 0.31
WIDTH_INNER_SPI_BRACT	5.49 ± 0.65	5.42 ± 0.65	4.43 ± 0.77	4 ± 0.37	4.12 ± 0.84	5.64 ± 0.8	4.49 ± 0.58	6.23 ± 0.73	5.84 ± 0.72	4.86 ± 0.49	4.89 ± 0.94	5.55 ± 0.83
WIDTH_INTER_INV_BRACT	4.34 ± 1.18	4.79 ± 0.9	3.31 ± 0.74	3.43 ± 0.6	3.59 ± 0.75	4.11 ± 0.96	4.83 ± 0.9	5.26 ± 1.14	3.91 ± 0.79	4.15 ± 0.58	3.44 ± 0.73	4.44 ± 0.76
WIDTH_OUT_INV_BRACT	2.98 ± 0.68	3.19 ± 0.59	2.36 ± 0.37	2.7 ± 0.58	2.78 ± 0.65	2.45 ± 0.7	3.41 ± 0.6	3.15 ± 0.8	2.37 ± 0.7	3.14 ± 0.59	2.69 ± 0.59	2.98 ± 0.53
WIDTH_OUTER_SPI_BRACT	5.14 ± 0.43	5.79 ± 0.63	4.65 ± 0.65	4.2 ± 0.48	4.1 ± 0.6	5.72 ± 0.89	4.39 ± 0.56	5.77 ± 0.97	5.7 ± 0.74	4.81 ± 0.47	4.58 ± 1.03	5.49 ± 0.7
WIDTH_OUTER_SPI_BRATLE	2.39 ± 0.34	2.9 ± 0.42	2.31 ± 0.29	2.41 ± 0.29	2.5 ± 0.48	2.37 ± 0.43	2.86 ± 0.47	2.7 ± 0.77	2.26 ± 0.75	2.7 ± 0.45	2.68 ± 0.74	2.83 ± 0.39
WIDTH_SUM_LEAF	2.17 ± 0.63	4.36 ± 1.03	3.47 ± 1.28	2.94 ± 0.97	3.78 ± 1.11	2.86 ± 0.56	3.64 ± 1.01	2.52 ± 0.68	2.52 ± 0.67	3.88 ± 1.43	2.27 ± 0.68	3.14 ± 0.69
WIDTH_WIN_LEAF	3.11 ± 0.78	5.16 ± 0.85	4.66 ± 0.93	3.85 ± 1.23	5.43 ± 1.6	4.63 ± 0.65	5.63 ± 1.74	3.78 ± 0.68	3.31 ± 0.79	4.23 ± 1.4	NA	4 ± 0.83

Table S5. Median and inter-quartile range of quantitative discrete characters for the twelve populations studied. Characters as in Table 2 and populations as in Table 1. * = type locality.

**Character**	**AA**	**BO**	**BR**	**FB***	**GA**	**LA***	**LL***	**MB***	**MC**	**MP**	**PS**	**TV**
SCAP_NUM	2 ± 3.5	3,5 ± 1.75	5,5 ± 5.25	8 ± 10.5	2 ± 2.75	5,5 ± 7.25	15 ± 32.5	8 ± 8.5	5 ± 4.5	6,5 ± 5	4 ± 5	4 ± 3.75
N_INV_BRACT	11 ± 2.75	13 ± 2	13 ± 3.25	16 ± 3.5	14 ± 4	13 ± 2.25	15 ± 2	13 ± 3	14,5 ± 2.5	14 ± 4	10 ± 2	13.5 ± 2.5
N_SUM_VEINS	3 ± 0	6 ± 2	7 ± 0	5 ± 2	7 ± 2	3 ± 0.5	7 ± 2	3 ± 0	3 ± 0	7 ± 2	7 ± 2	7 ± 2
N_WIN_VEINS	3 ± 0	5 ± 2.25	7 ± 2	3 ± 2	6 ± 2	3 ± 2	5 ± 4	3 ± 1	3 ± 2	5 ± 2	NA	5 ± 2

Table S6. Mean and standard deviation of the karyological indices in *Armeria arenaria* from the twelve studied populations. Indices names as in the main text; *n* = number of studied metaphasic plates. Population codes as in Table 1. * = type locality.

Population	*n*	THL	M_{CA}	CV_{CL}	CV_{CI}
AA	4	42.98 ± 3.98	27.42 ± 2.19	17.16 ± 1.69	14.44 ± 0.63
BO	5	40.52 ± 3.89	26.25 ± 0.88	17.12 ± 3.22	14.82 ± 2.68
BR	6	37.08 ± 3.41	25.57 ± 0.85	16.15 ± 2.16	12.49 ± 1.99
FB*	4	42.04 ± 2.22	23.51 ± 2.86	16.26 ± 4.79	10.74 ± 2.04
GA	6	45.37 ± 7.80	26.61 ± 1.65	16.86 ± 4.01	13.54 ± 1.89
LL*	4	42.04 ± 2.68	25.73 ± 2.51	16.12 ± 2.34	15.65 ± 3.47
LA*	4	48.37 ± 6.33	28.78 ± 1.21	18.84 ± 2.09	15.22 ± 2.58
MC	4	48.88 ± 4.76	27.12 ± 2.43	18.22 ± 1.29	15.93 ± 2.52
MB*	4	46.16 ± 6.75	27.85 ± 2.48	18.76 ± 2.44	15.08 ± 0.85
MP	7	37.64 ± 5.22	22.78 ± 1.76	17.19 ± 2.62	14.05 ± 2.83
PS	7	41.71 ± 5.38	25.01 ± 2.79	18.31 ± 1.97	15.88 ± 2.22
TV	6	39.54 ± 1.89	23.44 ± 2.37	16.82 ± 4.03	12.06 ± 2.75

Table S7. List of molecular markers and their primers.

Marker	Primer (F-R)	Sequence (5′→3′)	References
ITS	JK14	GGA GAA GTC GTA ACA AGG TTT CCG	[50]
	JK12	CCA AAC AAC CCG ACT CGT AGA CAG C	
trnF-trnL	trnF (GAA) IGS_f	GGT TCA AGT CCC TCT ATC CC	[51]
	trnF (GAA) IGS_r	ATT TGA ACT GGT GAC ACG AG	
trnH-psbA	psbA	GTT ATG CAT GAA CGT AAT GCT C	
	trnH (GUG)	CGC GCA TGG TGG ATT CAC AAT CC	
trnQ-rps16	trnQ (UUG)	GCG TGG CCA AGY GGT AAG GC	[52]
	rpS16x1	GTT GCT TTY TAC CAC ATC GTT T	
rpl32-trnL	trnL (UAG)	CTG CTT CCT AAG AGC AGC GT	
	rpL32-F	CAG TTC CAA AAA AAC GTA CTT C	

Table S8. PCR settings used.

Step	Phase	*ITS*		*trnQ-rps16*		*trnH-psbA; trnL-F*		*trnL-rpl32*	
		T°C	t	T°C	t	T°C	t	T°C	t
1	Initial denaturation	95	3 min	95	3 min	95	3 min	95	3 min
2	Denaturation	95	30 s	95	30 s	95	30 s	95	30 s
(35 cycles)	Annealing	62	30 s	53	30 s	55	30 s	53	30 s
	Extension	72	90 s	72	60 s	72	35 s	72	90 s
3	Extension	72	7 min	72	3 min	72	60 s	72	7 min

Table S9. Number of phylogenetically informative characters per marker and for the concatenated matrix.

Marker	Length (bp)	Consensus length (bp)	N. phylogenetically informative characters
ITS	804	804	11
ITS1	194	194	2
5,8S	156	156	--
ITS2	256 (454)[1]	256 (454)	7 (2)
trnF-trnL	311	311	4
trnH-psbA	320	320	7
trnL-rpl32	566-570	571	13
trnQ-rps16	329-330	330	1
Concatenated matrix	----	2337	36

Table S10. IDL test results ($\alpha < 0.05$).

	ITS	*trnF-trnL*	*trnH-psbA*	*trnL-rpl32*	*trnQ-rps16*	*Plastid*
ITS	NA	0.06	0.1	0.01	1.0	0.01
trnF-trnL		NA	0.82	1.0	0.73	-
trnH-psbA			NA	0.37	0.07	-
trnL-rpl32				NA	0.43	-
trnQ-rps16					NA	-
Plastid						NA
trnF+trnH	0.1					
trnF+trnH+trnQ	0.09					
trnH+trnL+trnQ	0.01					

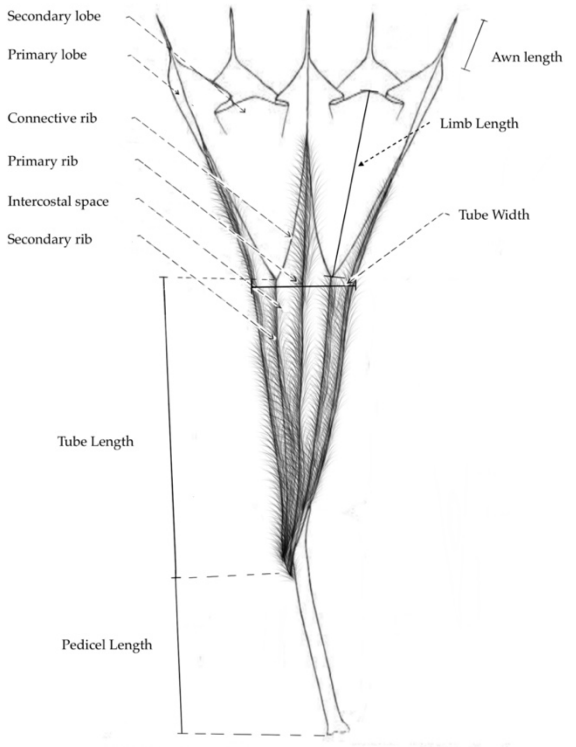

Figure S1. Schematic drawing of a calyx and related morphometric characters measured. (Figure from Lawrence [30], modified).

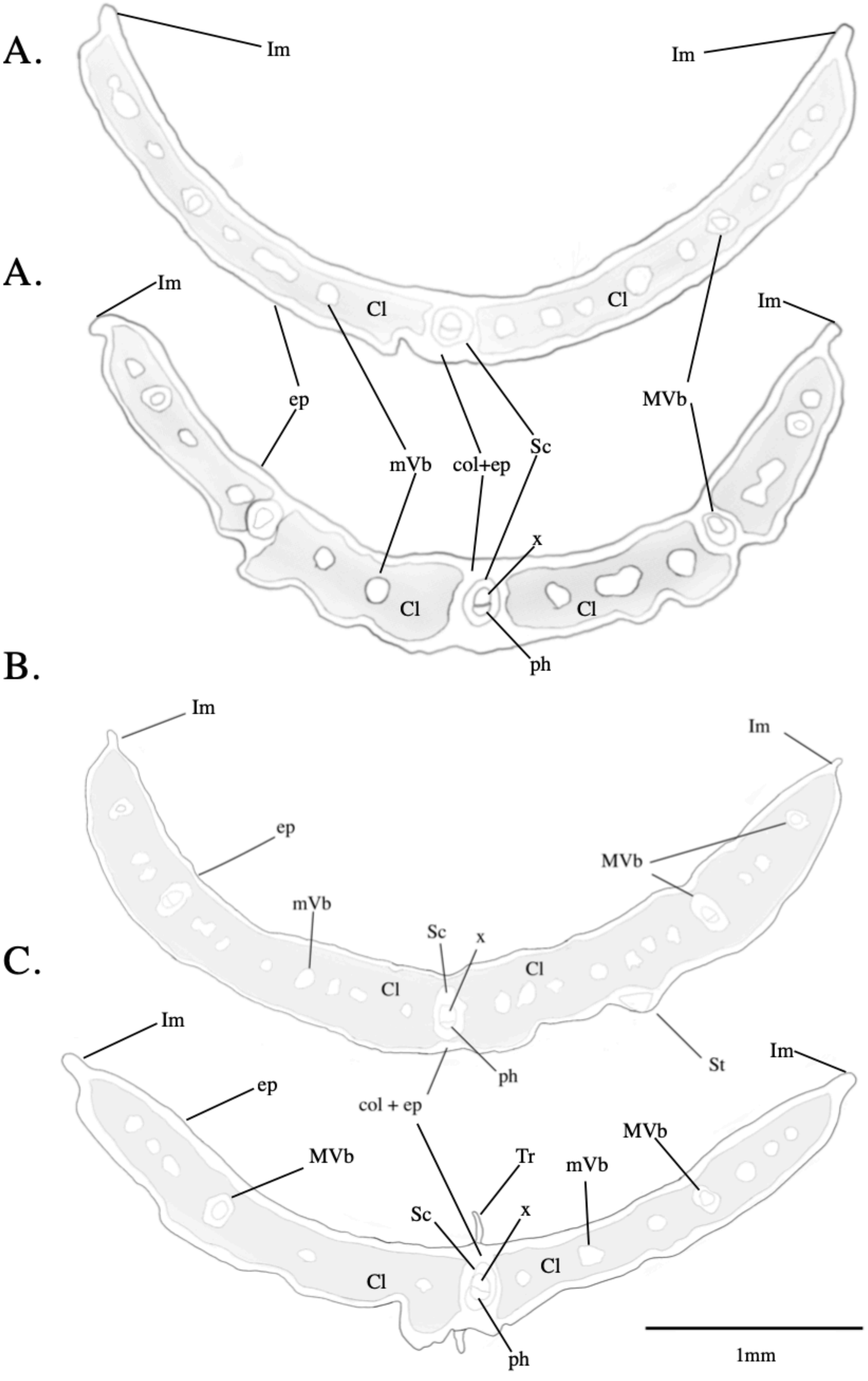
A.
Im
Im
A.
Im
Cl
Cl
Im
ep
MVb
mVb
col+ep
Sc
x
Cl
Cl
B.
ph
Im
Im
ep
MVb
mVb
Sc
x
C.
Cl
Cl
St
ph
Im
Im
col + ep
ep
MVb
Tr
mVb
MVb
Sc
x
Cl
Cl
ph
1mm

[continued]

D.

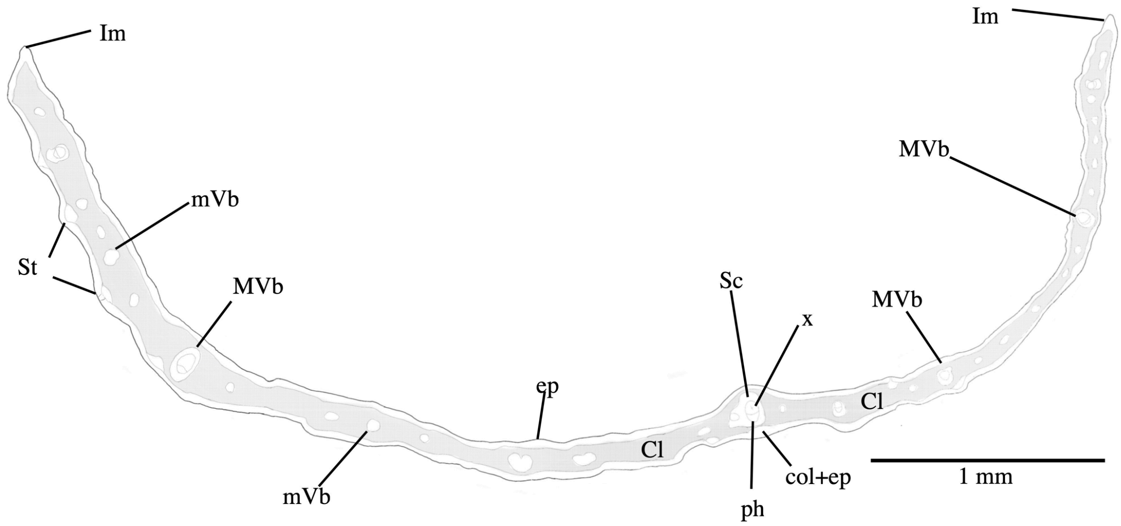

Figure S2. Schematic drawings of selected summer leaf cross sections from the type localities of the four putative *Armeria arenaria* subspecies. A. Two cross sections from LA (individuals LA13 and LA06), the topotypical population of *Armeria arenaria* subsp. *marginata*. B. Cross section from FB (individual FB14), the topotypical population of *A. arenaria* s.str. C. Cross section from MB (MB10), the topotypical population of *Armeria arenaria* subsp. *apennina*. D. Cross section from LL (individual LL10), the topotypical population of *Armeria arenaria* subsp. *praecox*. Population codes as in Table 1. Im = hyaline margin, MVb = main vascular bundles, mVb = minor vascular bundles, ep = epidermis, Cl = chlorenchyma, x = xylem, ph = phloem, Sc = sclerenchyma, col = collenchyma, St = Stomata, Tr = trichome.

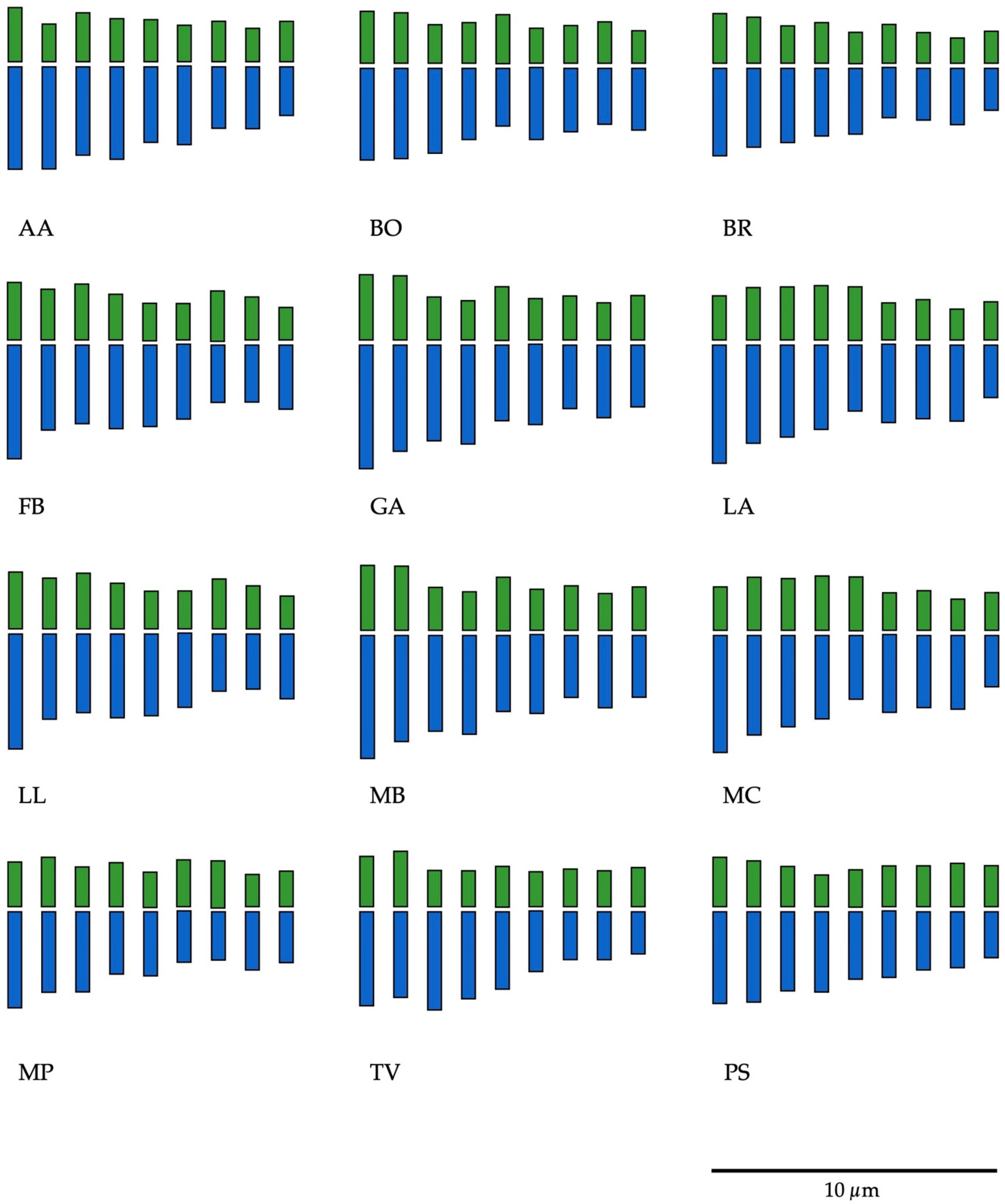

Figure S3. Haploid idiograms (x = 9) of the twelve populations studied. Green = short arm; Blue = long arm. Scale bar is indicated at the bottom right. Population codes as in Table 1.

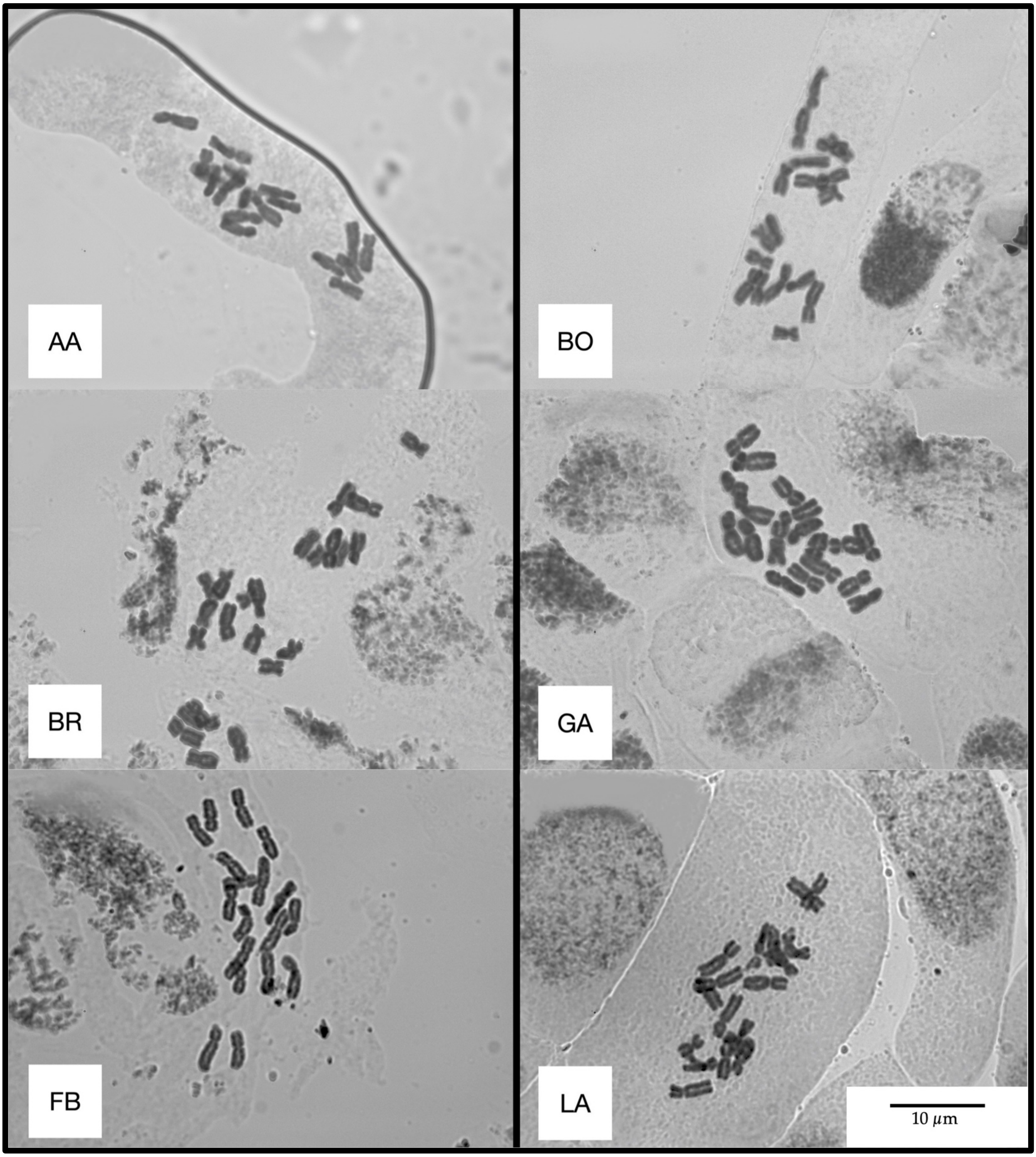

[continued]

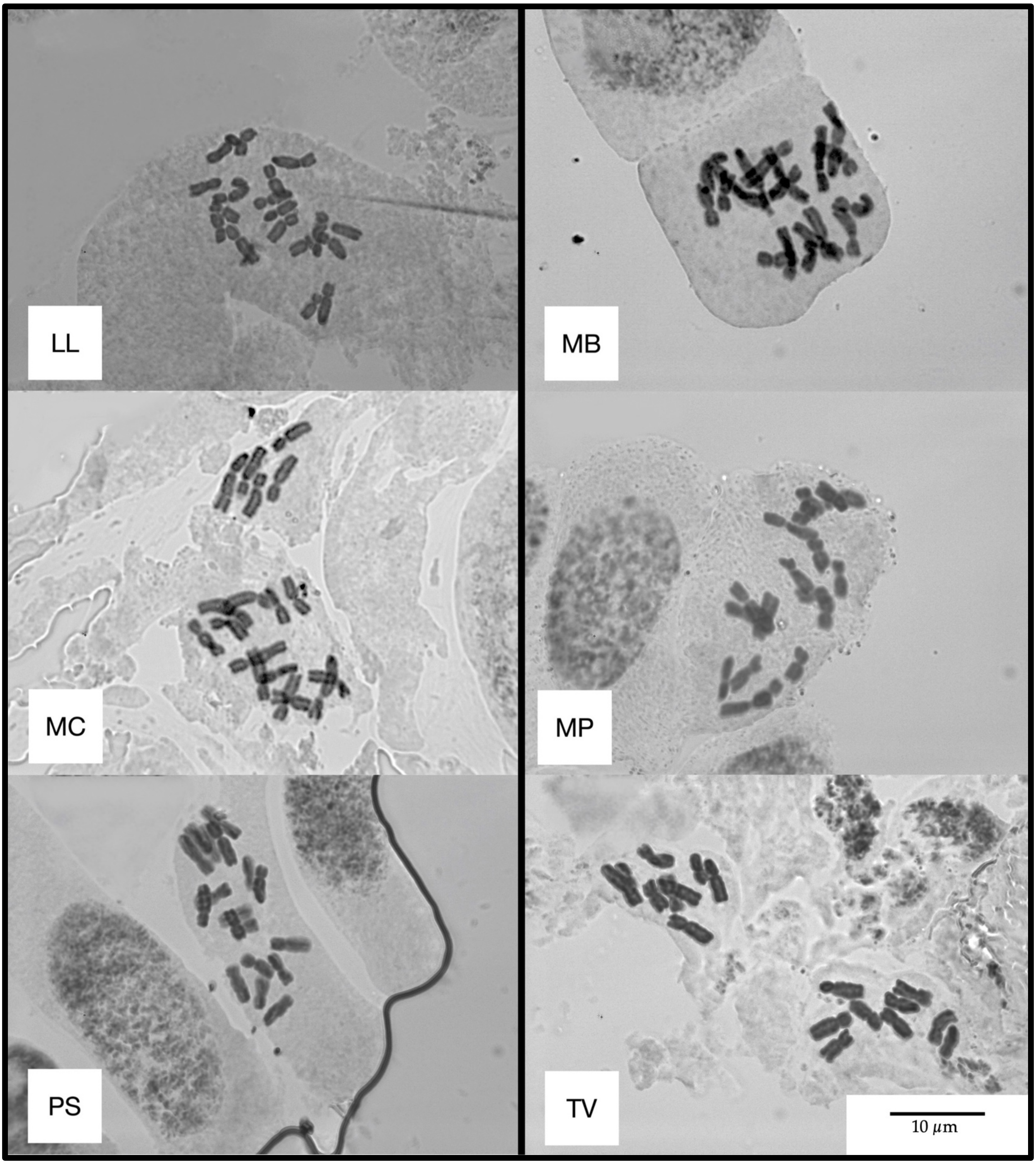

Figure S4. Selected metaphasic plates of the twelve populations studied. Scale bar is indicated at the bottom right. Population codes as in Table 1.

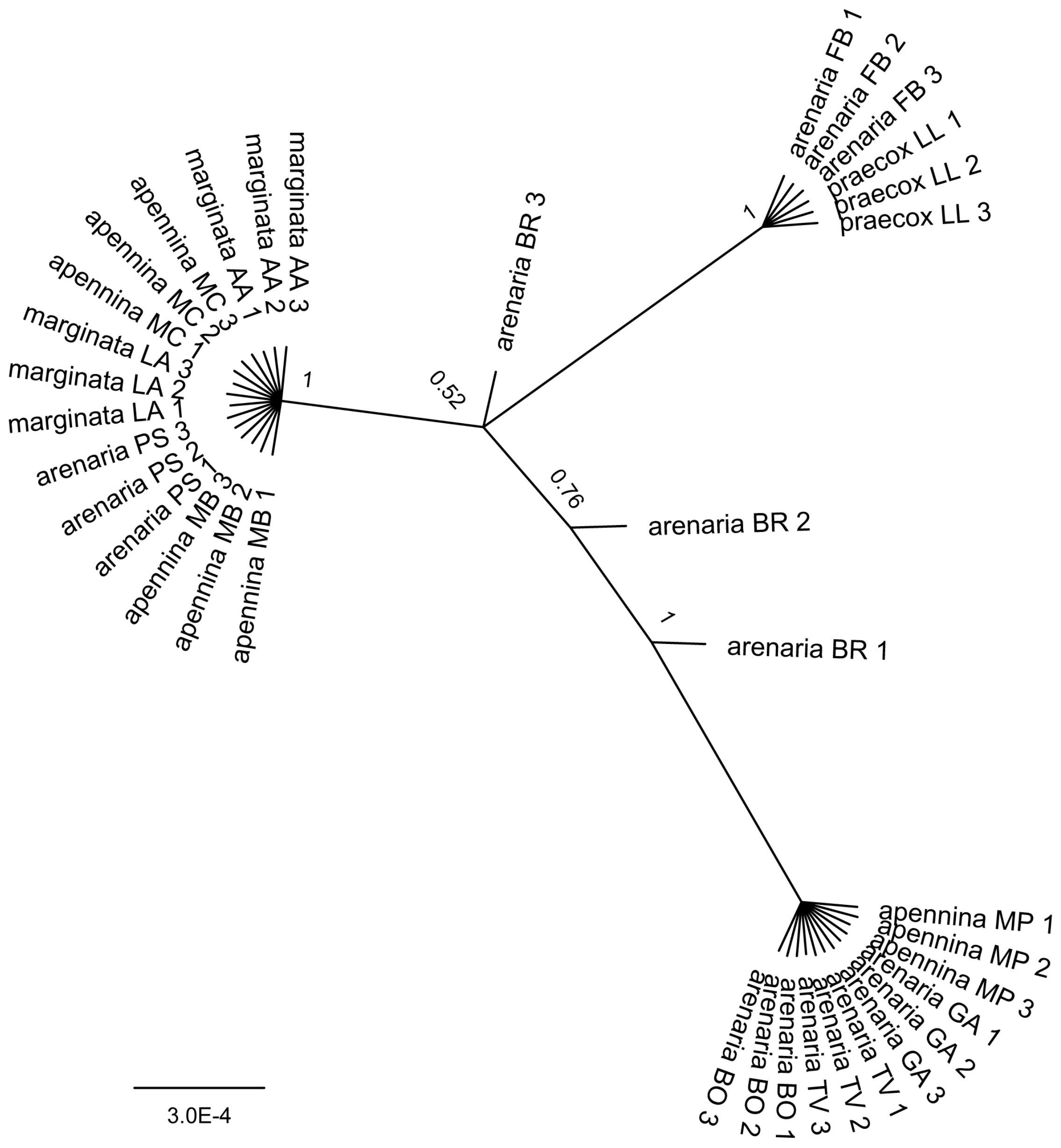

Figure S5. Bayesian unrooted consensus phylogenetic tree of ITS matrix. AA = Apuan Alps, N Apennines; BO = Bobbio, N Apennines; BR = Brusson, Pennine Alps; FB = Fontainebleau, Île-de-France; GA = Gambolò, West Po Valley; LA = Libro Aperto; N Apennines; LL = Le Lauzet, Dauphiné Alps; MB = Marmagna–Braiola, N Apennines; MC = Monte Cusna, N Apennines; MP = Monte Prinzera, N Apennines; PS = Piana di Salmezza, Lombard Prealps; TV = Terme di Valdieri, Maritime Alps. Further population details are provided in Table 1 and Figure 6 of the main text.

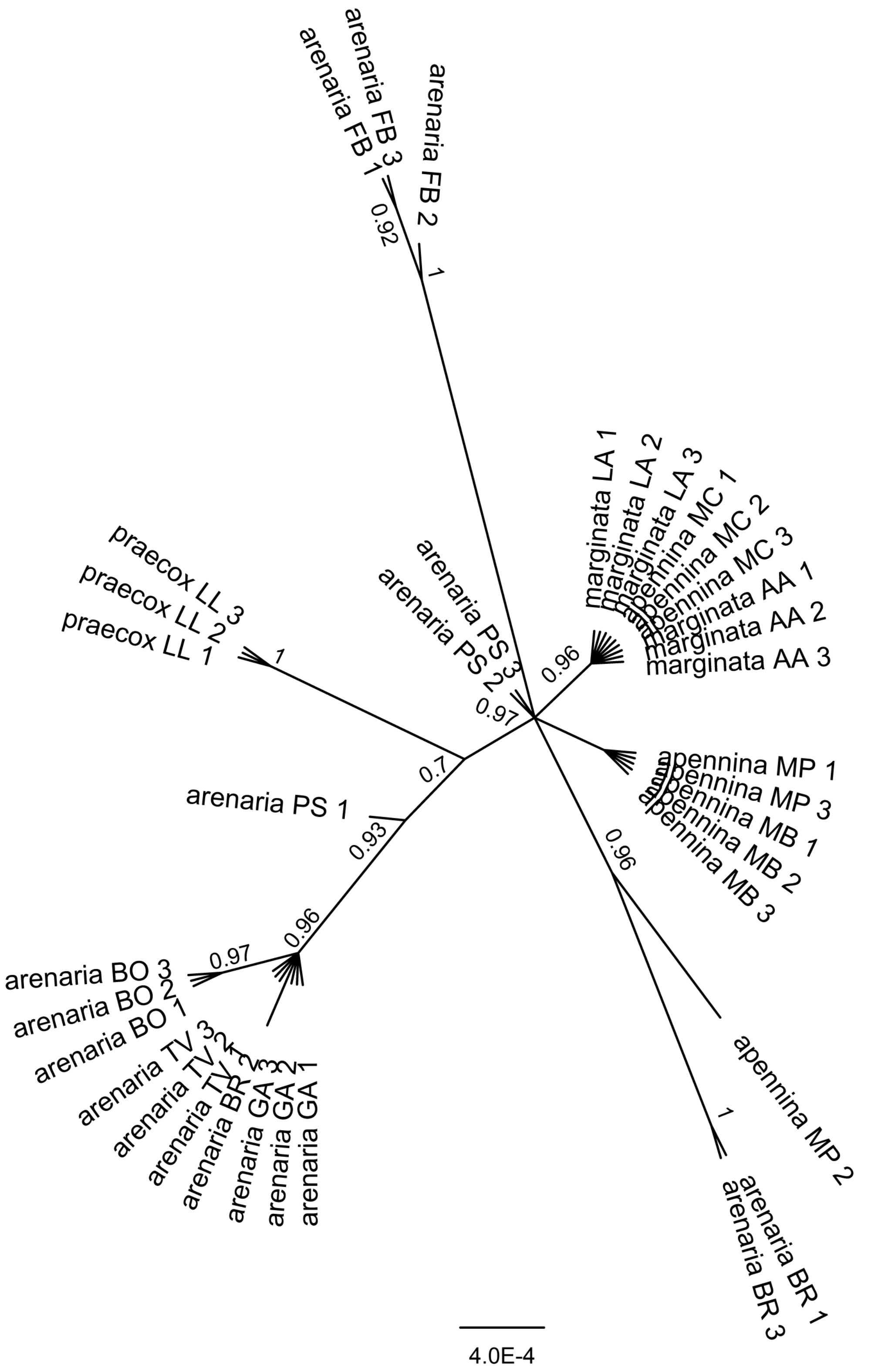

Figure S6. Bayesian unrooted consensus phylogenetic tree of plastid matrix. AA = Apuan Alps, N Apennines; BO = Bobbio, N Apennines; BR = Brusson, Pennine Alps; FB = Fontainebleau, Île-de-France; GA = Gambolò, West Po Valley; LA = Libro Aperto; N Apennines; LL = Le Lauzet, Dauphiné Alps; MB = Marmagna–Braiola, N Apennines; MC = Monte Cusna, N Apennines; MP = Monte Prinzera, N Apennines; PS = Piana di Salmezza, Lombard Prealps; TV = Terme di Valdieri, Maritime Alps. Further population details are provided in Table 1 and Figure 6 of the main text.
